# Recurrent Systemic Embolization in a Patient With Aortic Valve Amyloidosis

**DOI:** 10.1111/echo.70241

**Published:** 2025-07-15

**Authors:** Annalisa Caputo, Amedeo Pergolini, Luigi Monticelli, Enrico Natale, Antonio Lio, Maria Cristina Macciomei, Giulia D'Amati, Martina Leopizzi, Carla Manzara

**Affiliations:** ^1^ Department of Cardiovascular and Respiratory Science Sapienza University of Rome Rome Italy; ^2^ Department of Cardiac Surgery and Heart Transplantation S. Camillo Forlanini Hospital Rome Italy; ^3^ Department of Cardiovascular Science ASL Roma 2 Rome Italy; ^4^ Department of Cardiovascular Science S. Camillo Forlanini Hospital Rome Italy; ^5^ Unit of Pathology S. Camillo Forlanini Hospital Rome Italy; ^6^ Department of Radiological Oncological and Pathological Sciences Sapienza University Rome Italy

**Keywords:** amylodosis, aortic valve, y, echocardiography

## Abstract

A rare case of a 47‐year‐old woman with recurrent thromboembolic events, including STEMI and lower limb ischemia, diagnosed with aortic valve thrombosis caused by isolated aortic valve amyloidosis in the setting of overlapping antiphospholipid syndrome.

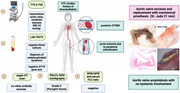

1

A 47‐year‐old Caucasian female with a history of tobacco smoking, one spontaneous abortion, and a prior episode of thrombophlebitis during pregnancy presented to the Emergency Department with complaints of chest pain and dyspnea. An electrocardiogram revealed a diagnosis of posterior ST‐elevation myocardial infarction (STEMI). Urgent coronary angiography revealed a thrombotic occlusion of the first obtuse marginal branch, which was treated with thrombus aspiration followed by implantation of a drug‐eluting stent.

The patient was immediately started on dual antiplatelet therapy and anticoagulation with enoxaparin.

Transthoracic and transesophageal echocardiography performed subsequently revealed a myxomatous appearance of the aortic valve, with significant thickening and restricted leaflet mobility, leading to moderate stenosis (mean gradient of 34 mmHg on continuous Doppler). Endocarditis was ruled out based on the absence of vegetations and negative serial blood cultures (Figure 1A).

**FIGURE 1:**
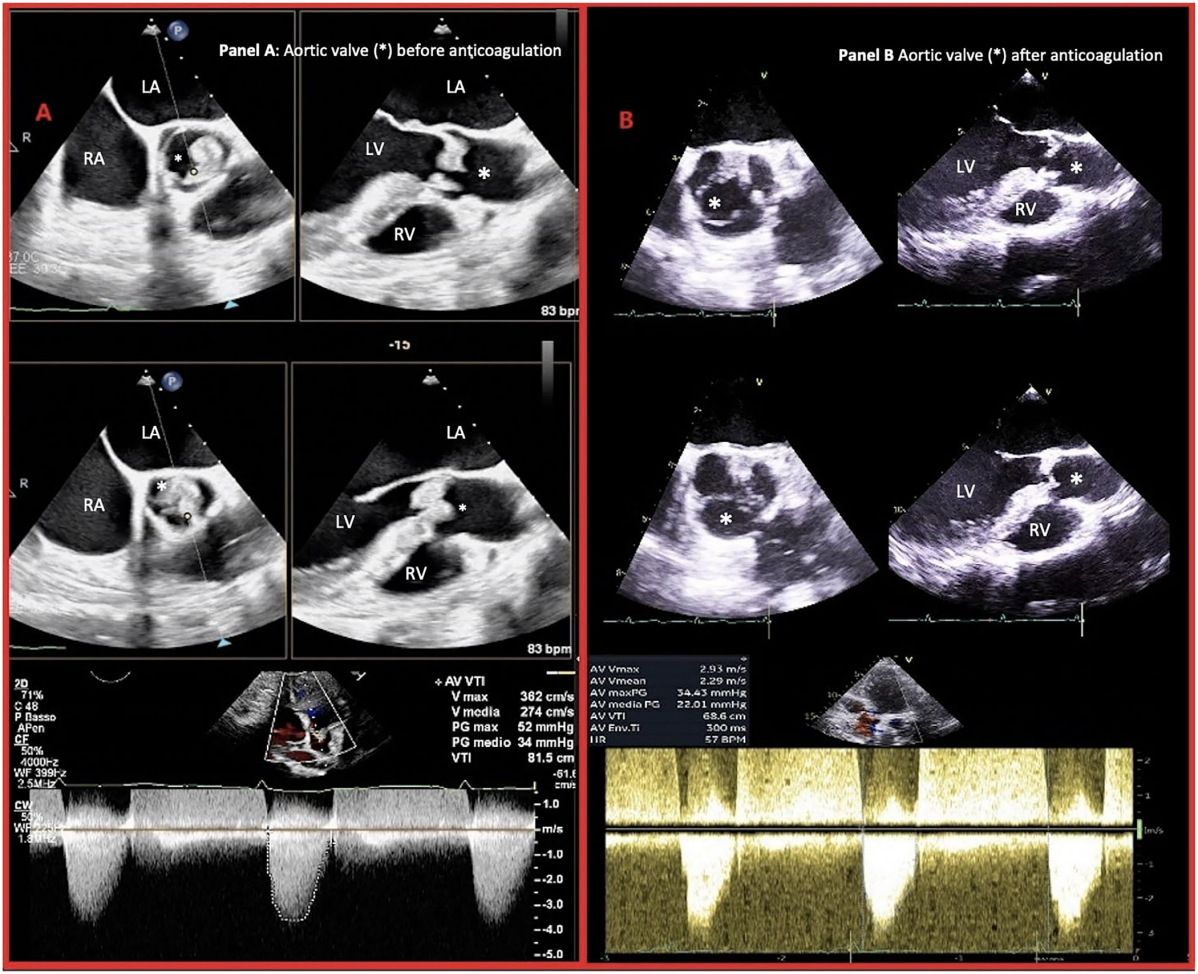
Transoesophageal echocardiography performed before (Panel A) and after (Panel B) anticoagulation.

During hospitalization, the patient experienced acute ischemia of the right lower limb, attributed to an occlusion of the right external iliac artery, which was successfully treated with a Fogarty embolectomy. Histological examination of the extracted thrombus confirmed the presence of thrombotic material without signs of infection. Continuous telemetry monitoring and subsequent Holter EKG monitoring did not reveal atrial fibrillation episodes. A subsequent angio‐CT scan excluded other embolic sources, leading to the conclusion that both the coronary and peripheral thrombotic events originated from the aortic valve. Given the patient's history of recurrent thromboembolic events, a thrombophilia panel was conducted, confirming a diagnosis of antiphospholipid syndrome. After 1 week of anticoagulation therapy with enoxaparin, repeat echocardiography showed persistent but reduced thickening of the aortic valve with a lower mean transvalvular gradient (24 mmHg) (Figure 1B). A multidisciplinary team determined that surgical aortic valve replacement was warranted. The patient underwent successful aortic valve replacement with a 21 mm St. Jude mechanical prosthesis and was started on treatment with a vitamin K antagonist, aspirin, and clopidogrel. Clopidogrel was discontinued after one week

Examination of the excised native valve revealed no signs of endocarditis or thrombosis (Figure 2A).

**FIGURE 2:**
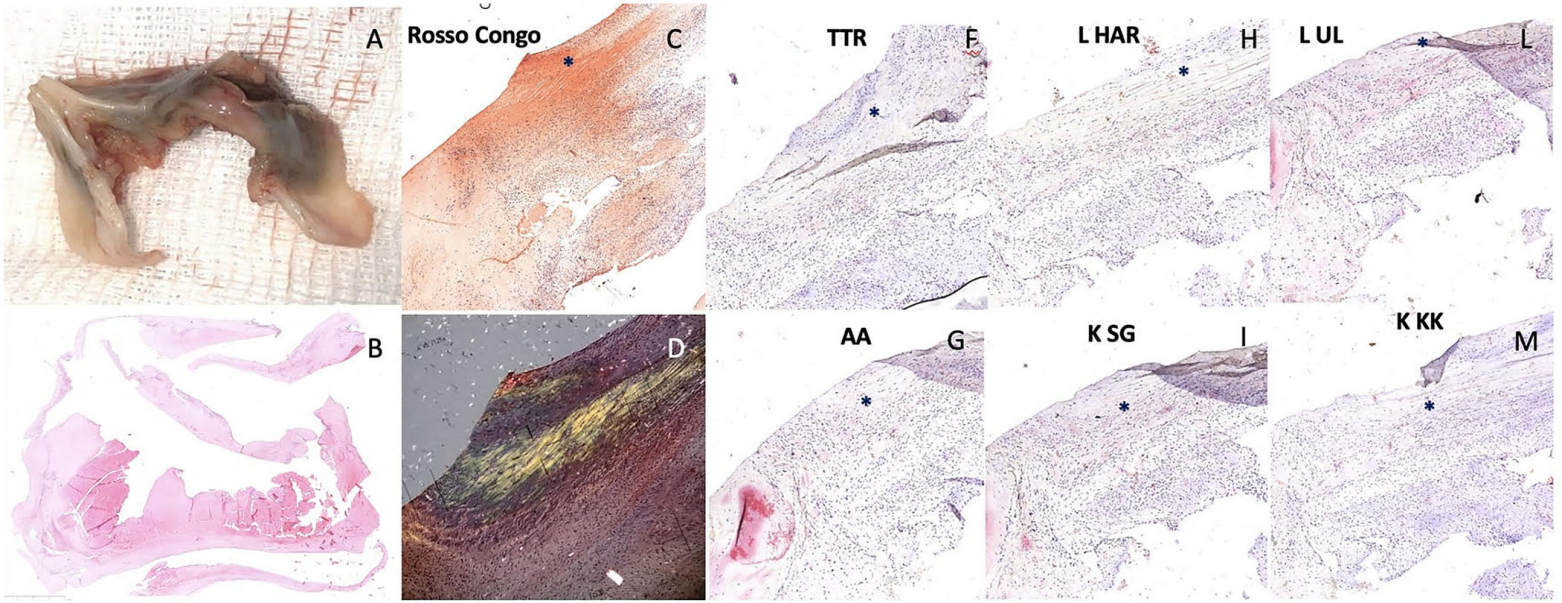
Excised native valve (A). Hematoxylin‐Eosin staining of valve tissue revealing deposits of an eosinophilic amorphous substance (B). Congo red staining (C) revealing apple‐green birefringence under polarized light (D) confirming amyloid. Immunohistochemical staining using anti‐ATTR, anti‐AL lambda, anti‐ALk and anti‐AA antibodies revealed no significant immunoreactivity with amyloid deposits (F–M).

Histopathological analysis demonstrated dense connective tissue covered by endothelium, with extensive deposits of amorphous acellular material consistent with amyloid. Hematoxylin and eosin staining showed disruption of the normal cusp architecture by an eosinophilic amorphous substance (Figure 2B). Congo red staining (exhibited apple‐green birefringence under polarized light (Figure 2D). Immunohistochemical staining using anti‐ATTR, anti‐ALλ, anti‐ALκ, and anti‐AA antibodies revealed no significant immunoreactivity within the amyloid deposits (Figure 2F–M).

Speckle tracking echocardiography did not reveal suggestive signs of myocardial amyloidosis, although posterior wall necrosis was evident (Figure 3). A 99mTc‐DPD scintigraphy scan showed no systemic uptake (Perugini Score Grade 0) (Figure 4), and tests for clonal dyscrasia were negative, including serum free light chain (FLC) ratio, serum (SPIE), and urine (UPIE) protein electrophoresis with immunofixation. Genetic testing ruled out mutations in the ATTR gene, suggesting a rare form of isolated valvular amyloidosis. The patient was discharged in good clinical condition 2 weeks post‐surgery, on treatment with VKA and cardioaspirin. At the 12‐month follow‐up, she remains asymptomatic, with stable prosthetic valve function and no recurrence of thromboembolic events. A study of 136 cardiac valves found amyloid microdeposition in 51% of aortic and 34% of mitral valves with sclerotic or calcific lesions, independent of patient age. Immunohistochemistry identified an unknown amyloid protein distinct from recognized systemic or localized amyloid types [1]. While light‐chain amyloidosis (AL) with myocardial involvement frequently presents with left heart valve thickening, cases of exclusive valvular amyloidosis without myocardial or systemic involvement are exceedingly rare [2]. Previous reports have described amyloid‐infiltrated aortic valves leading to coronary embolism, as in this case [3, 4].

**FIGURE 3:**
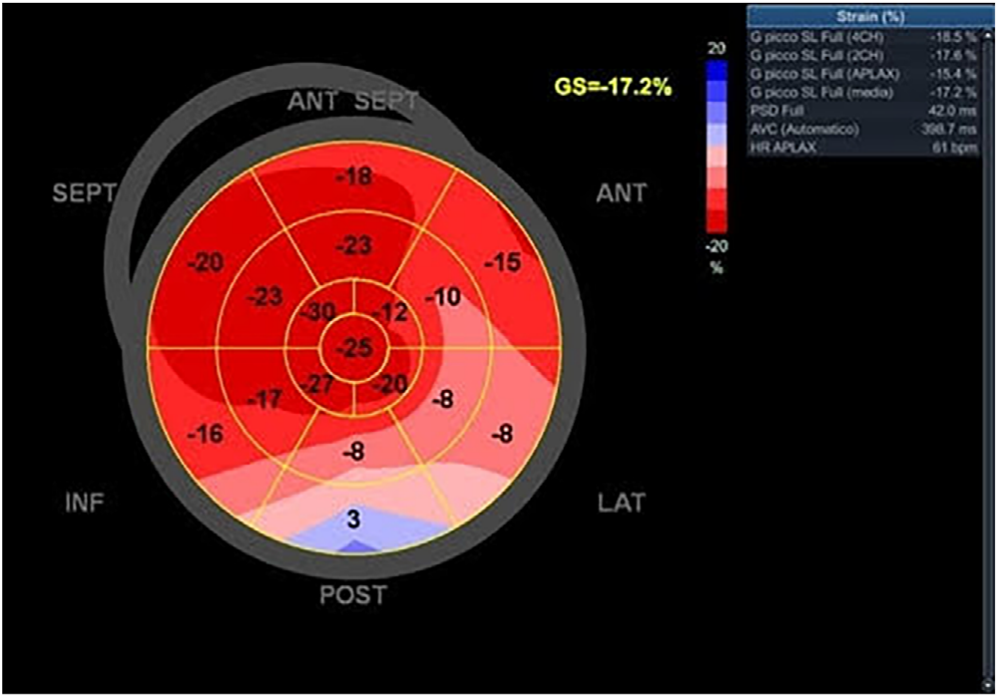
Global Longitudinal Strain (GLS) analysis performed using speckle tracking echocardiography; apical segments display preserved strain, suggestive of absence of myocardial involvement from cardiac amyloidosis, while posterior segments exhibit markedly reduced deformation.

**FIGURE 4:**
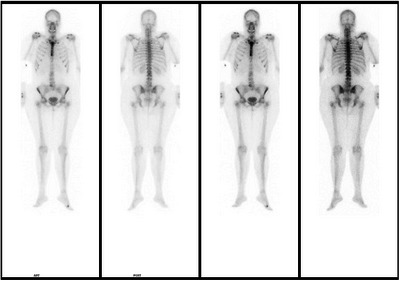
99mTc‐DPD scan showing no systemic uptake of the tracer (Perugini Grade 0).

This case illustrates a rare presentation of isolated valvular amyloidosis complicated by leaflet thrombosis in a patient with antiphospholipid syndrome. Recurrent thromboembolic events, supported by obstetric and thrombotic history and positive antibody testing, were compounded by amyloid‐induced valvular damage, ultimately necessitating valve replacement despite an initial response to anticoagulation.

Echocardiography was instrumental in guiding diagnosis and management, but definitive identification required histological examination. Further research is needed to characterize this novel form of amyloid deposition in cardiac valves.

## Supporting information




**Supporting File 1**: echo70241‐vid‐0001.avi.

**Supporting File 2**: echo70241‐vid‐0002.avi.

## Data Availability

The data that support the findings of this study are available from the corresponding author upon reasonable request.
